# Supplementary material for: Probing embedded topological modes in bulk-like GeTe-Sb2Te3 heterostructures
Source: Sci Rep. 2020 Dec 11;10:21806. doi: 10.1038/s41598-020-76885-7 (PMC7732994; doi:10.1038/s41598-020-76885-7)
Supplement: Supplementary file 1 — Supplementary information. [file 41598_2020_76885_MOESM1_ESM.pdf]

Supplementary Material: Probing embedded topological modes in bulk-like GeTe-Sb_2Te_3 heterostructures

Hisao Nakamura[1,2,*], Johannes Hofmann[3,4,*], Nobuki Inoue[5], Sebastian Koelling[6], Paul M. Koenraad[6], Gregor Mussler[7], Detlev Grützmacher[7], and Vijay Narayan[8,*]

[1]CD-FMat, National Institute of Advanced Industrial Science and Technology (AIST), 1-1-1 Umezono, Tsukuba Central 2, Tsukuba, Japan
[2]Department of Materials Science and Metallurgy, University of Cambridge, 27 Charles Babbage Road, Cambridge CB3 0FS, United Kingdom
[3]Department of Applied Mathematics and Theoretical Physics, University of Cambridge, Centre for Mathematical Sciences, Cambridge CB3 0WA, United Kingdom
[4]TCM Group, Cavendish Laboratory, University of Cambridge, Cambridge CB3 0HE, United Kingdom
[5]RIKEN Center for Computational Science, 7-1-26 Minatojima-minami, Cyuo-ku, Kobe, Hyogo 650-0047, Japan
[6]Eindhoven University of Technology, 5600 MB Eindhoven, The Netherlands
[7]Peter Grünberg Institute (PGI-9), Forschungszentrum Jülich, 52425 Jülich, Germany
[8]Department of Physics, University of Cambridge, J. J. Thomson Avenue, Cambridge CB3 0HE
[*]hs-nakamura@aist.go.jp, jbh38@cam.ac.uk, vn237@cam.ac.uk

ABSTRACT

This Supplementary Materials contains materials characterisation of the three-layer heterostructures considered in Sections 3 and 4 of the main manuscript. Here we show that our simulations and continuum model consider an experimentally relevant structure where there is a small degree of intermixing between otherwise GeTe and Sb_2Te_3-rich layers.

1 X-ray diffraction analysis

X-ray (XRD) measurements were accomplished with a high-resolution Bruker D8 diffractometer, employing a scintillator detector with a mechanical slit of 3 mm. Figure 1 depicts a $2\theta/\theta$ measurement of a Sb_2Te_3/GeTe/$Sb_2 3Te_3$ sample. The scan was carried out in a range from $2\theta = 3\check{}140°$ with angular resolution of 0.1°. Each data point was measured for 10 s. Besides the Si substrate peaks, signatures of the Sb_2Te_3 and GeTe epilayers are found. The reflections of the epilayers' peaks are always (0,0,n), evidencing that both, Sb_2Te_3 and GeTe, are single-crystalline films with the z-axis in growth direction.

2 Atom Probe Tomography

Atom Probe Tomography (APT) was carried out using a LEAP 4000X-HR from Cameca. The system is equipped with a laser-generating picosecond pulses at a wavelength of 355 nm. For the analysis, all samples were cooled down to a temperature of 20 K. The experimental data are collected at a laser pulse rate of 65 – 125 kHz using laser powers between 1.75 and 5 pJ per pulse. The data are reconstructed using IVAS 3.6.

In order to create the APT depth profile, the reconstructed volume is sliced into 0.1 nm thick discs along the z-axis. The elemental atoms/ions in each disk are then used to calculate the concentrations of the respective element at the particular position along the z-/depth axis.

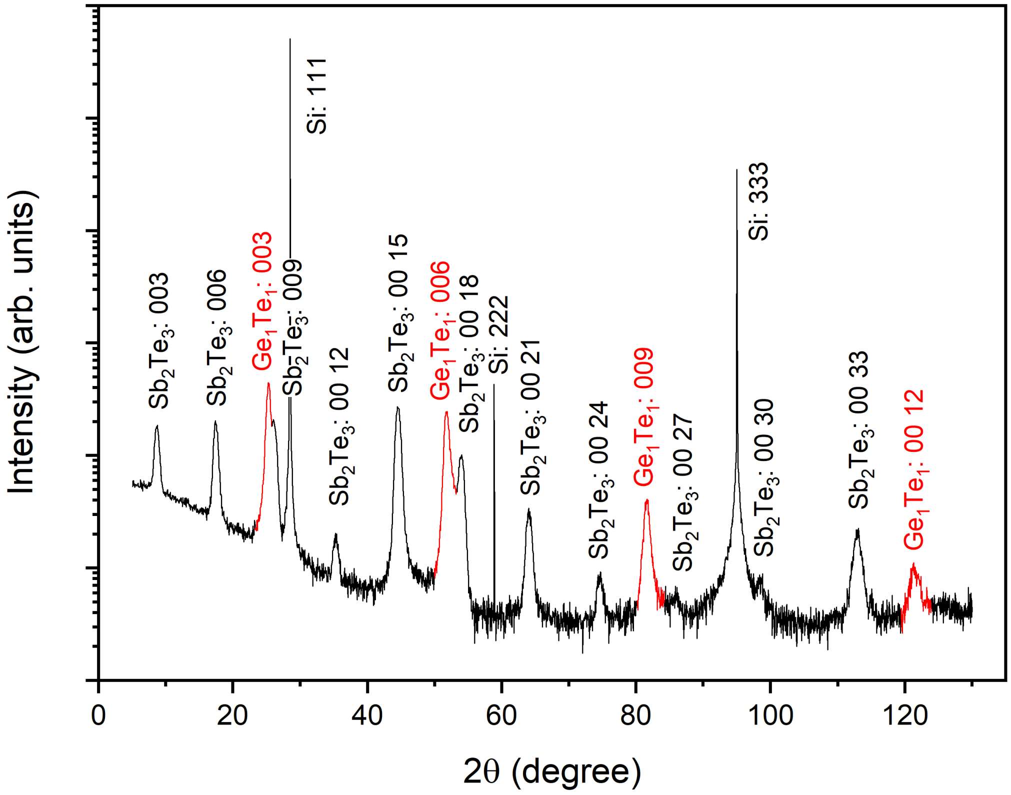

Supplementary Figure 1. XRD pattern of the Sb_2Te_3-GeTe heterostructure.

3 First Principles Calculation

In our calculations, we use the SIESTA[1] and Smeagol[2] program packages and adopt the double zeta plus polarization function (DZP) level basis set. Our results are consistent with the experimental findings presented in the previous section. To evaluate the band offset and identify the SG interfacial electronic states, we make use of the non-equilibrium Green's function technique[3] combined with DFT (NEGF-DFT). Since NEGF-DFT satisfies semi-infinite boundary condition, our results are free from artificial size effects, which are often problematic in slab model calculations. NEGF-DFT calculations were carried out using the Smeagol program package[2,4]. We adopt an exchange correlation (XC) functional of the van der Waals (vW) correction, DF2[5], for total energy calculations and use the local density approximation (LDA) to determine the band structure in NEGF-DFT calculations, where spin-orbit (SO) interaction are included.

At room temperature, bulk GeTe crystallises in a disordered rock salt (dRk) structure and Sb_2Te_3 crystallises in a rhombohedral layered (Rh) structure[6]. Hence, the conventional hexagonal cell can be taken as the unit cell for both, where the (111) direction of the rock salt corresponds to the (0001) in the conventional hexagonal cell. We set the c-axis to the (0001) direction and define the z-coordinate along the c-axis. The SG interface plane is then perpendicular to the (0001) direction. In the conventional hexagonal cell, both GeTe (dRk) and Sb_2Te_3 (Rh) have a CBA/CBA/CBA/... stacking, where A, B, and C represent monolayers of Sb_2Te_3 or GeTe. In other words, the bulk unit cell of Sb_2Te_3 and GeTe consists

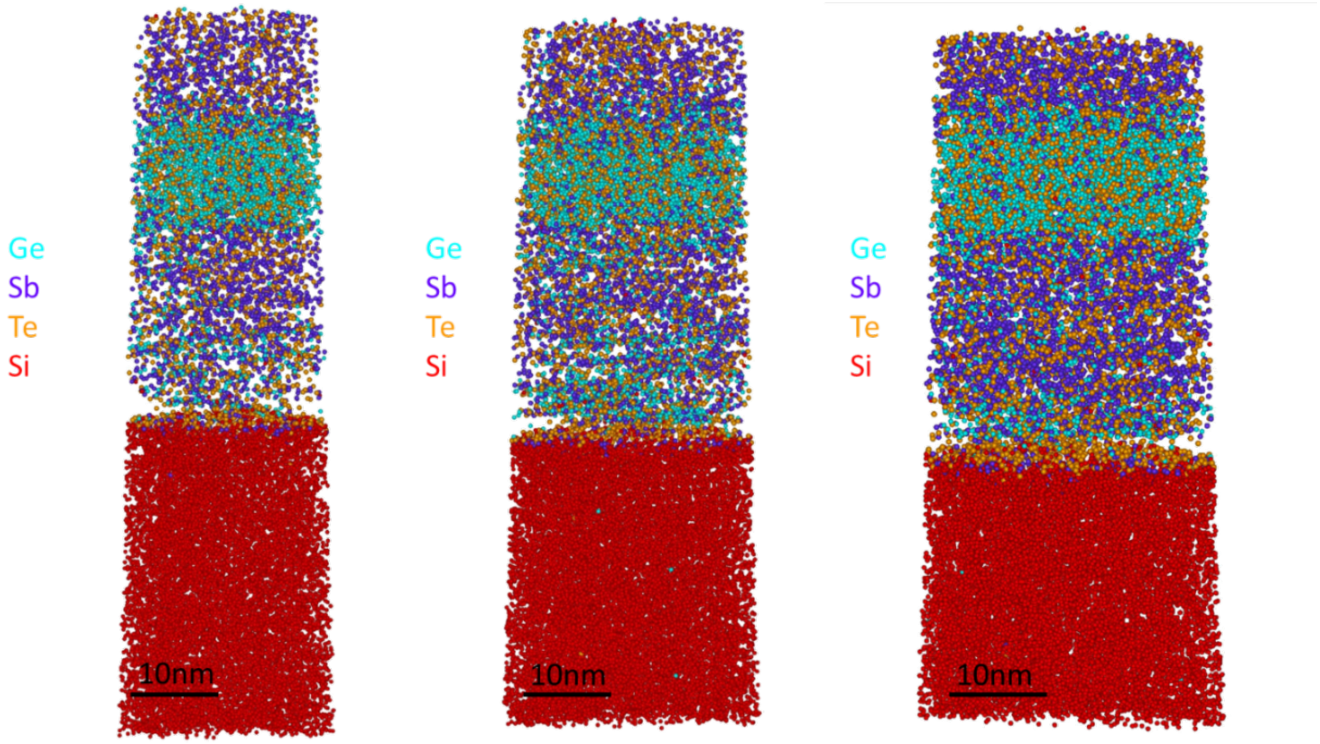

Supplementary Figure 2. Atom Probe Tomography reconstruction of three different samples taken from a wafer of Sb_2Te_3-GeTe-Sb_2Te_3 heterostructure.

of three monolayers, which may be denoted as $(Sb_2Te_3)_3$ and $(GeTe)_3$, respectively[6,7]. The experimental values of the lattice constants are $a_0 = b_0 = 4.26$ and $c_0 = 30.75$ for Sb_2Te_3 (Rh) and $a_0 = b_0 = 4.17$ and $c_0 = 10.90$ for GeTe (dRk)[7]. Thus, there is a very small lattice mismatch at the SG interface. For simplicity, we fix the lattice constant as $a_0 = b_0 = 4.25$ for both of Sb_2Te_3 and GeTe (as well as the SGS tri-layer) in our computational models and then allow all atoms in the cell to relax to their atomic positions. The atomic structures are shown in Figs. 3(a) and 3(b), respectively.

4 Four-band model calculations

This section discusses the description of the heterostructure band structure using the envelope function formalism that uses effective four-band models for the separate bulk phases, the parameters of which were presented in the main text.

For a slab geometry with an interface in the xy-plane, we loose translational invariance in the z direction. The Hamiltonian of a heterostructure takes the form of Eq. (3) in the main text with z-dependent coefficients $A_1(z), A_2(z), \ldots$, i.e.,

$$\hat{H}(k_x, k_y, k_z) \to \hat{H}(k_x, k_y, -i\partial_z). \tag{1}$$

Note that in the heterostructure, the second z-derivative must be written in a manifestly symmetric form to ensure a hermitian Hamiltonian, i.e., we replace[8]

$$-B_2 k_z^2 \to \frac{d}{dz} B_2(z) \frac{d}{dz}. \tag{2}$$

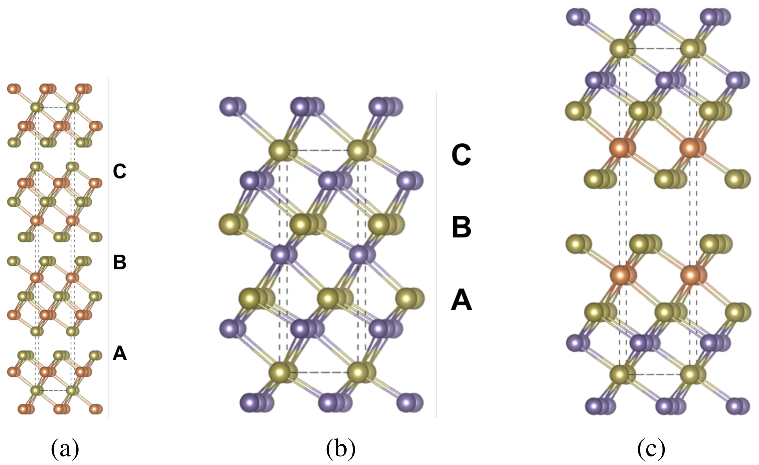

Supplementary Figure 3. Crystal structures of bulk (a) Sb_2Te_3 (rhombohedral), (b) GeTe (disordered rock salt), and (c) GST225 (Kooi structure). The purple, dark yellow and orange balls represent Ge, Te, and Sb atoms, respectively. All structures are represented in the conventional hexagonal cell, which is indicated by dotted lines. The unit cells of Sb_2Te_3 and GeTe consist of three stacked monolayers of Sb_2Te_3 and GeTe, which are labeled by A, B, and C, respectively.

Eigenstates that solve $\hat{H}\Psi(z) = E\Psi(z)$ depend on the parallel momentum $k_\parallel$ as well as the z coordinate. We consider solutions within a layer with the ansatz[8]

$$\Psi_{\alpha\beta}(z,E) = \Psi_{\alpha\beta}(E)e^{\beta\lambda_\alpha(E)z}, \tag{3}$$

where λ_α is a complex-valued function of E and $\alpha,\beta = \pm$. The eigenstate equation for $\Psi(z,E)$ reads

$$\begin{pmatrix} -\lambda_\alpha^2 D_- + L_1 & -i(\beta\lambda_\alpha)B_0 & 0 & A_0 k_- \\ -i(\beta\lambda_\alpha)B_0 & -\lambda_\alpha^2 D_+ + L_2 & A_0 k_- & 0 \\ 0 & A_0 k_+ & -\lambda_\alpha^2 D_- + L_1 & i(\beta\lambda_\alpha)B_0 \\ A_0 k_+ & 0 & i(\beta\lambda_\alpha)B_0 & -\lambda_\alpha^2 D_+ + L_2 \end{pmatrix} \Psi_{\alpha\beta}(E) = E\Psi_{\alpha\beta}(E) \tag{4}$$

with

$$\begin{aligned} D_\pm &= C_1 \mp M_1 \\ L_1 &= C_0 + M_0 + (C_2 + M_2)k_\parallel^2 \\ L_2 &= C_0 - M_0 + (C_2 - M_2)k_\parallel^2. \end{aligned} \tag{5}$$

The value of λ_α is obtained in closed analytical form by solving Eq. (5), which gives[9,10]

$$\lambda_\alpha^2(E) = -\frac{F}{2D_+D_-} + \alpha\frac{\sqrt{R}}{2D_+D_-}, \tag{6}$$

where

$$\begin{aligned} F &= B_0^2 + D_+(E - L_1) + D_-(E - L_2) \\ R &= F^2 - 4D_+D_-[(E - L_1)(E - L_2) - A_0^2 k_+ k_-]. \end{aligned} \tag{7}$$

For each value of $\beta\lambda_\alpha(E)$, there are two linearly independent eigenvectors:

$$\Psi_{\alpha\beta 1}(E) = \begin{pmatrix} E+\lambda_\alpha^2 D_+ - L_2 \\ -i(\beta\lambda_\alpha)B_0 \\ 0 \\ A_0 k_+ \end{pmatrix}, \quad \Psi_{\alpha\beta 2}(E) = \begin{pmatrix} A_0 k_- \\ 0 \\ i(\beta\lambda_\alpha)B_0 \\ E+\lambda_\alpha^2 D_- - L_1 \end{pmatrix}. \tag{8}$$

The full solution in each layer is composed of these eight eigenvectors. Eight boundary conditions then determine the relative magnitude of the eigenvectors (with the overall magnitude fixed by the normalization) and the energy E. The condition on E follows from the determinant of the boundary conditions.

It remains to determine boundary conditions. First, we require $\Psi = 0$ at the two boundaries of the slab (eight boundary conditions). Second, at each interface, we impose the continuity of the wavefunction and the current (which gives four boundary conditions per interface). This can be written as

$$\begin{aligned} \Psi(-\varepsilon) &= \Psi(+\varepsilon) \\ \mathscr{M}\Psi(-\varepsilon) &= \mathscr{M}\Psi(+\varepsilon), \end{aligned} \tag{9}$$

where the flux operator $\mathscr{M}$ is obtained by integrating the Hamiltonian across the interface

$$\mathscr{M} = \begin{pmatrix} (C_1+M_1)\frac{\partial}{\partial z} & iB_0 & 0 & 0 \\ iB_0 & (C_1-M_1)\frac{\partial}{\partial z} & 0 & 0 \\ 0 & 0 & (C_1+M_1)\frac{\partial}{\partial z} & -iB_0 \\ 0 & 0 & -iB_0 & (C_1-M_1)\frac{\partial}{\partial z} \end{pmatrix}. \tag{10}$$

4.1 Continumm model applied to an Sb_2Te_3 slab

Here we consider a single Sb_2Te_3 (Rh) film to estimate the strength of the interlayer coupling. The single slab geometry has been solved in Refs.[9–11]. Results of this calculation using Sb_2Te_3 parameters are shown in Fig. 4. Figure 4(a) shows the position of the valence band maximum and the conduction band minimum at the Γ point as a function of the slab thickness L_s (orange lines). These states are surface states that are localised at the edge of the slab and gap out due to the intralayer coupling between the surface modes. The plot also includes the first bulk bands as blue lines. As for Bi_2Se_3[11], there is an oscillating exponential decay of the gap with increasing slab thickness L_s. The inset illustrated the band structure near the Γ point for two values of the thickness $L_s = 5$ nm and 10 nm, where the gapped surface Dirac state is indicated by the orange lines and bulk bands by blue lines. Figure 4(b) shows the gap as a function of slab thickness L_s. As is apparent from the figure, surface states on the left and right edges are sufficiently decoupled for $L_s = 15$ nm, at which point the energy gap caused by intralayer coupling between the surface states is negligible (less than 0.01 eV). Furthermore, as is apparent from Fig. 4(a), for thicknesses above 15 nm, the upper and lower energies of the surface states at Γ point coalesce to 0.14 eV below the Fermi level of Sb_2Te_3. The Sb_2Te_3 film is thus "bulk-like" in in this regime.

References

1. Soler, J. M. *et al.* The SIESTA method forab initioorder-nmaterials simulation. *J. Physics: Condens. Matter* **14**, 2745, DOI: 10.1088/0953-8984/14/11/302 (2002).
2. Rocha, A. R. *et al.* Spin and molecular electronics in atomically generated orbital landscapes. *Phys. Rev. B* **73**, 085414, DOI: 10.1103/PhysRevB.73.085414 (2006).

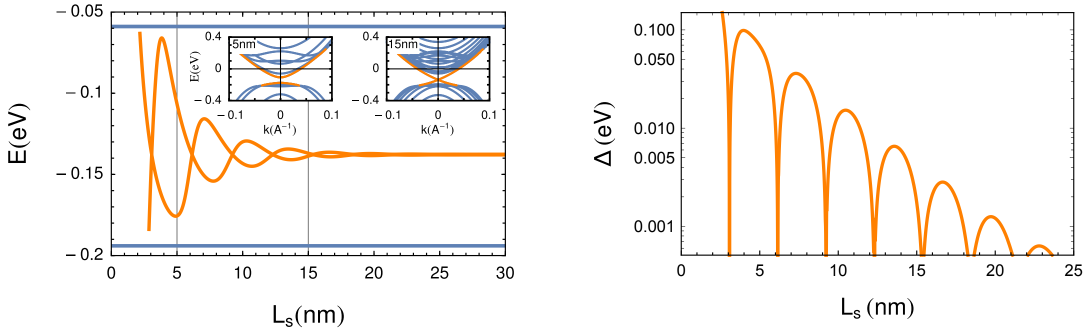

(a) (b)

Supplementary Figure 4. Band structure of a single Sb_2Te_3 slab as obtained from the four-band model. (a) Energy levels of the surface states at the Γ point as a function of slab thickness L_s. (b) Difference of the two surface states energies at the Γ point in the bulk band gap. When the slab thickness is larger than 15 nm, the two surface states are almost degenerate.

3. Datta, S. *Electronic Transport in Mesoscopic Systems* (Cambridge University Press, 1997).

4. Rungger, I. & Sanvito, S. Algorithm for the construction of self-energies for electronic transport calculations based on singularity elimination and singular value decomposition. *Phys. Rev. B* **78**, 035407, DOI: 10.1103/PhysRevB.78.035407 (2008).

5. Klimeš, J., Bowler, D. R. & Michaelides, A. Chemical accuracy for the van der waals density functional. *J. Physics: Condens. Matter* **22**, 022201, DOI: 10.1088/0953-8984/22/2/022201 (2009).

6. Da Silva, J. L. F., Walsh, A. & Lee, H. Insights into the structure of the stable and metastable $(GeTe)_m(Sb_2Te_3)_n$ compounds. *Phys. Rev. B* **78**, 224111, DOI: 10.1103/PhysRevB.78.224111 (2008).

7. Nonaka, T., Ohbayashi, G., Toriumi, Y., Mori, Y. & Hashimoto, H. Crystal structure of gete and ge2sb2te5 meta-stable phase. *Thin Solid Films* **370**, 258, DOI: https://doi.org/10.1016/S0040-6090(99)01090-1 (2000).

8. Bastard, G. & Brum, J. Electronic states in semiconductor heterostructures. *IEEE J. Quantum Electron.* **22**, 1625, DOI: 10.1109/JQE.1986.1073186 (1986).

9. Zhou, B., Lu, H.-Z., Chu, R.-L., Shen, S.-Q. & Niu, Q. Finite size effects on helical edge states in a quantum spin-hall system. *Phys. Rev. Lett.* **101**, 246807, DOI: 10.1103/PhysRevLett.101.246807 (2008).

10. Shan, W.-Y., Lu, H.-Z. & Shen, S.-Q. Effective continuous model for surface states and thin films of three-dimensional topological insulators. *New J. Phys.* **12**, 043048, DOI: 10.1088/1367-2630/12/4/043048 (2010).

11. Linder, J., Yokoyama, T. & Sudbø, A. Anomalous finite size effects on surface states in the topological insulator bi_2se_3. *Phys. Rev. B* **80**, 205401, DOI: 10.1103/PhysRevB.80.205401 (2009).
